# Dysphagia Affecting Quality of Life in Cerebellar Ataxia—a Large Survey

**DOI:** 10.1007/s12311-020-01122-w

**Published:** 2020-03-13

**Authors:** M. Rönnefarth, N. Hanisch, A. U. Brandt, A. Mähler, M. Endres, F. Paul, Sarah Doss

**Affiliations:** 1 Department of Neurology, Charité–Universitätsmedizin Berlin, Humboldt-Universität zu Berlin, and Berlin Institute of Health, Charitéplatz 1, 10117 Berlin, Germany; 2 NeuroCure Clinical Research Center, Charité–Universitätsmedizin Berlin, Charitéplatz 1, 10117 Berlin, Germany; 3 Experimental & Clinical Research Center, Charité–Universitätsmedizin Berlin and Max Delbrück Center for Molecular Medicine, Lindenberger Weg 80, 13125 Berlin, Germany; 4 Movement Disorders Section, Department of Neurological Sciences, University of Nebraska Medical Center, 4242 Farnam Street, 68131 Omaha, NE USA

**Keywords:** Dysphagia, Cerebellar ataxia, Health-related quality of life, Spinocerebellar ataxia, Nutrition

## Abstract

Dysphagia is a common symptom in neurodegenerative disorders and is generally associated with increased mortality. In the clinical care setting of ataxia patients, no systematical and standardized assessment of dysphagia is employed. Its impact on patients’ health-related quality of life is not well understood. To assess the impact of dysphagia in ataxia patients on diet, body weight, and health-related quality of life. We conducted a large survey using self-reported questionnaires for swallowing-related quality of life (Swal-QOL) and a food frequency list in combination with retrospective clinical data of 119 patients with cerebellar ataxia treated in the neurological outpatient clinic of a large German university hospital. Seventeen percent of ataxia patients suffered from dysphagia based on the Swal-QOL score. Less than 1% of all patients reported dysphagia as one of their most disabling symptoms. Dysphagia was associated with unintentional weight loss (*p* = 0.02) and reduced health-related quality of life (*p* = 0.01) but did not affect individual nutritional habits (*p* > 0.05; Chi-squared test). Dysphagia is a relevant symptom in cerebellar ataxia. A systematic screening for dysphagia in patients with cerebellar ataxia would be desirable to enable early diagnosis and treatment.

## Introduction

Dysphagia is a common symptom in patients suffering from neurodegenerative disorders that affect motor control. In ataxia disorders, studies on prevalence, consequences, and prognostic implications of dysphagia are scarce. Cerebellar ataxia arises from different etiologies, classified as hereditary or sporadic. For spinocerebellar ataxia (SCA), the reported prevalence of dysphagia varies considerably from 6 to 74%, depending on several factors including the underlying genetic mutation with lower prevalence in pure cerebellar syndromes, e.g., in SCA 6 [1–5].

Symptoms in cerebellar ataxia comprise gait unsteadiness, limb ataxia, dysarthria, dizziness, and dysphagia [6]. While gait and speech disorders in chronic disease are known to deteriorate health-related quality of life (HRQOL) [7, 8], a link between dysphagia and HRQOL has not been investigated yet. Neurogenic dysphagia impedes food consumption and may lead to weight loss, malnutrition, and dehydration [4, 9, 10]. More drastically, it may cause life-threatening aspiration and pneumonia with increased morbidity and mortality [8, 11, 12]. A post mortem pathologic study reported neurodegeneration of the brainstem nuclei to be involved in ingestion as well as aspiration pneumonia to be the major cause of death in patients suffering from SCA-3 [13]. In SCA, every phase of the swallowing process (lingual, pharyngeal, and esophageal phase) can be impaired [14].

Reduced quality of life accompanies the progression of chronic neurological diseases, but it remains unclear which factors determine the individual quality of life in ataxia patients [7, 15].

To prevent complications, early detection of swallowing disorders is important. Furthermore, identification of influencing factors on HRQOL is crucial for individualized and specific treatment. It remains unclear if swallowing disorders in cerebellar ataxia affect patients’ nutritional habits and are related to complications such as weight loss and lower body weight.

To discern factors influencing HRQOL and dysphagia-related consequences in daily life, we conducted a written survey in a clinically well-characterized large patient cohort suffering from progressive cerebellar ataxia of various origins. To screen for swallowing deficits and their influence on quality of life, we used the Swal-QOL, a validated, wildly used, subjective assessment tool [16]. The food-frequency list (FFL) was used to assess individual nutritional habits [17].

## Materials and Methods

### Study Design and Patients

For this cross-sectional observational study, we screened patients regularly attending the ataxia outpatient clinic of a large university hospital (Charité-Universitätsmedizin Berlin, Germany) from November 2011 to April 2014. Inclusion criteria were progressive ataxia not related to an acute event as defined by an experienced clinical assessor and willingness to participate in the study. We excluded patients with dementia or progressive cognitive decline. During routine clinical care visits, we assessed clinical parameters and—if indicated—genetic testing. Patients identified by the above-mentioned procedure (*n* = 190) received questionnaires and patient information together with the written informed consent by regular mail. Seventy-one percent of the questionnaires were answered and returned (*n* = 133). After exclusion of datasets with missing informed consent form (12) and incomplete data (2), questionnaires with complete Swal-QOL were included in the analysis (two excluded).

Table 1 refers to patient characteristics in the study cohort. We classified ataxia as hereditary, sporadic, or unknown. Patients suffering from hereditary ataxia had either positive genetic testing or a family member with genetically confirmed hereditary ataxia or strong clinical suspicion of hereditary ataxia based on pedigree, clinical presentation, and exclusion of other etiologies. Of the hereditary ataxias, 42 were autosomal dominant (28 patients with SCA 1–3, SCA 6, or SCA 17; 12 patients with SCA 11, 14, 27, or 28; two patients with Episodic Ataxia type 2 (EA2)), three recessive (one patient with ataxia telangiectasia and two patients classified as recessive according to pedigree analysis), and one mitochondrial ataxia. Among the sporadic ataxias, diagnosis of sporadic ataxia with onset in adulthood (SAOA) was established for 33 patients and criteria for multiple system atrophy with predominant cerebellar ataxia (MSA-C) were fulfilled in nine patients [18]. Other causes were autoimmune (*n* = 9) and toxic related to alcohol abuse (*n* = 6). In 16 patients classified as unknown, genetic testing for SCA 1–3, SCA 6, and Friedreich’s ataxia was negative and clinical signs for autonomic dysfunction were missing.

**Table 1:** Overview of patient characteristics

	All patients				Dysphagic				Non-dysphagic				MWU
	Mean	SD	Range	*N*	Mean	SD	Range	*N*	Mean	SD	Range	*N*	*p*
Age (years)	58.4	13.3	24–84	117	54.7	12.4	30–74	20	58.8	13.6	24–84	97	0.17
BMI (kg/qm)	25.4	5.1	16–51	117	23.6	5.2	16–34		25.7	5	16–51		0.15
Gender female (*n*)				59				10				49	0.97
Etiology of ataxia				117									0.35
Hereditary				46				9				37	
Sporadic				57				10				45	
Unknown				16				1				15	
Disease duration (years)	10.9	8.5	1–34	117	9.5	7.1	3–32		10.9	8.5	1–34		0.64
Age of onset (years)	47.4	14.6	4–73	117	45.5	12.5	27–68		47.9	15.1	4–73		0.31
Walking distance [Med; 1–4]	2	2.1	0–4	117	1	1.9	0–4		2	1.9	0–4		*0.001*
BDI II total score	13.7	9.6	1–4	117	22.3	9.1	1–4		12	8.7	1–4		*< 0.001*
BDI II classification													
1 (minimal)				64				2				61	
2 (mild)				27				7				20	
3 (moderate)				16				6				9	
4 (severe)				12				5				7	
FSS mean score	3.7	1.7	0–1	117	4.8	1.5	0–1		3.4	1.6	0–1		*0.001*
FSS classification													
0 (normal)				64				4				58	
1 (pathologic)				55				16				39	
Physical therapy (min/w)	52	44	0–240	117	69	54	0–240	17	49	40	0–120	70	0.12
Speech therapy (min/w)	23	37	0–120	117	35	33	0–120	13	21	37	0–120	29	*0.02*
Occupational therapy (min/w)	16	30	0–120	117	28	35	0–120	9	14	29	0–120	22	*0.04*

Walking distance scale: 0–4; median and variance reported

*BDI-II* Becks Depression Inventory, *FSS* Fatigue Severity Scale, *MWU* Mann-Whitney *U* test, *N* number of patients, *min/w* minutes per week, *SD* standard deviation

The local ethics committee at Charité-Universitätsmedizin Berlin (EA1/185/13) approved the study and it was conducted according to the 1964 Helsinki declaration in its currently applicable version. All participants gave written informed consent as described above.

#### General Design of the Survey

The survey included general questions about age, gender, body weight, and height. Patients were asked to name their most impairing symptoms and the age, when first symptoms were noticed [19]. A 5-point Likert scale assessed mobility, rating the maximal walking distance (if needed with the help of two persons): 0: < 200 m, 1: < 500 m, 2: < 1 km, 3: < 5 km, 4: > 5 km. To assess potential weight loss, patients reported if and how much weight they had lost in the past 12 months, if it had been unwanted (yes/no), if the patient had experienced reduced appetite (yes/no), and if the amount of food intake had been reduced in the last 12 months (yes/no). Ongoing therapy was defined by the time spent with physical, speech, and occupational therapies weekly. To assess overall impact of disease on quality of life, patients should report impairment of their subjective quality of life by the disease on an 11-point Likert scale: 0: strong impairment and 10: no impairment at all. To control for unspecific effects of pathologic fatigue and depressive symptoms, the self-reported Fatigue Severity Score (FSS, a score > 5 indicating pathologic fatigue) and Becks Depression Inventory (BDI-II, scores between 14 and 19 indicating mild, 20–28 indicating moderate, and above 29 indicating severe depression) were included in the questionnaire [20, 21].

#### Assessment of Dysphagia

The swallowing quality of life questionnaire (Swal-QOL) was used to assess dysphagia and related quality of life (QOL) using a validated German version of the questionnaire [16, 22, 23]. The Swal-QOL is a reliable and valid self-assessment tool covering all WHO ICF domains [24]. It comprises 44 items assessing 10 swallowing-related QOL domains: food selection, burden, mental health, social functioning, fear, eating duration, eating desire, communication, sleep, and fatigue. Additionally, 14 items focus on symptoms related to swallowing deficits; three questions evaluate nutritional intake (presence of percutaneous endoscopic gastrostomy (PEG) (yes/no), food, and liquid consistency) and one question addresses general health. Each item is rated on a 5-point Likert scale. After linear transformation, domain scores and a total score from 0 to 100 (100 indicating the most favorable state) are calculated. We used a seven-domain total score to identify patients with swallowing difficulties (scoring below 70). It comprises the first seven domains and reliably reflects swallowing-related QOL [16, 25]. Patients scoring below 70 are classified as dysphagic throughout the manuscript. Patients were allowed to fill out the questionnaire with the help of a proxy, which was reported by four patients (physical impairment, e.g., limb ataxia). Eleven patients were supported by a trustworthy person, e.g., a family member.

#### Assessment of Nutritional Habits

The self-reported Food-Frequency-List (FFL) of the MONICA project assessed individual nutritional habits, including 16 food categories based on the guidelines of the German Nutrition Society (DGE) [16, 17]. The mean weekly food intake frequency of, e.g., ‘meat (excluding sausage)’, ‘curds, yoghurt, sour milk’, ‘mineral water’, ‘fresh fruit’, and others were rated optimal, normal, or unfavorable. The FFL uses a six-point Likert scale to report frequency ranging from “almost daily intake” to “never”. Results are rated according to the method proposed by Winkler and coworkers [17]: each item is regarded separately, e.g., daily intake of mineral water is rated optimal whereas daily meat intake is rated unfavorable. With a maximum score of 32, 14–15 indicates normal food intake while lower scores are regarded as unfavorable and higher scores as optimal food intake habits.

### Data Analysis

We used SPSS Version 22 (IBM Corp, Armonk, NY, USA) for data analysis. Figures were created using MATLAB2008b with Statistics Toolbox Release 2012b (The MathWorks Inc., Natwick, MA, United States). To test for differences between dysphagic and non-dysphagic patients regarding diagnosis, gender, disease duration, therapies, body weight, FSS, and BDI-II mean scores, we used Mann-Whitney *U* test. For differences between dysphagic and non-dysphagic patients regarding weight loss, intention to lose weight, and reduction in appetite or food intake, we used Chi-squared test. For correlation between dysphagia and etiology, age, gender, disease duration, weight loss, body weight, mobility, quality of life, fatigue, depression, and nutritional habits, we calculated Spearman’s correlation coefficient.

We used multiple linear regression analysis to investigate the association of disease duration, etiology, mobility, FFL, Swal-QOL, FSS, and BDI-II classification on the variance of the self-reported QOL scale.

Significance level was established at *p* < 0.05 and adjusted for multiple testing for correlation analysis at *p* = 0.0045.

## Results

### Dysphagia as Self-reported Main Symptom Versus Swal-QOL

**Fig. 1:**
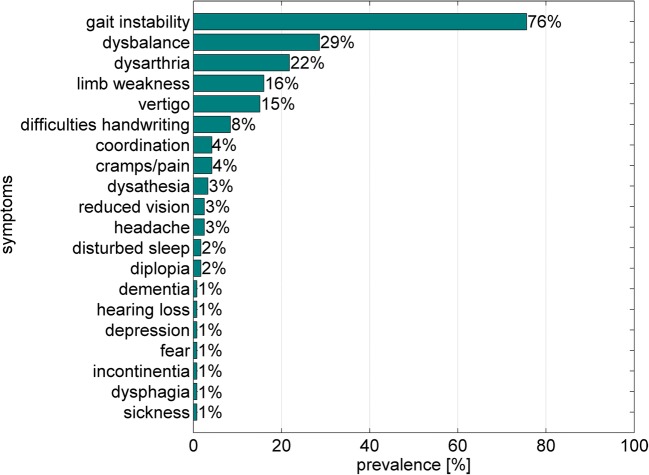
Most disabling self-reported symptoms affecting quality of life in all patients (multiple symptoms possible). Most patients reported gait instability. Only one patient reported dysphagia

A single patient reported dysphagia as part of his most disabling symptoms. In total, 20 different symptoms were mentioned (Fig. 1). This patient also reported pain, had a gastric feeding tube, and suffered from underweight (BMI = 17.6 kg/m^2^). Dysphagia was evident in his Swal-QOL total domain score (14.2) as well. This patient reported symptoms related to saliva and to pharyngeal but not oral phase in the questionnaire.

Furthermore, more than 75% of all patients reported gait instability and almost one third reported dysbalance. Each, dysarthria, vertigo, and limb weakness were reported by more than 10% of the patients. Patients reporting dysarthria as their most disabling symptom (*n* = 26) did not mention additional dysphagia and they did not show reduced body weight (mean BMI = 27 kg/m^2^ ± 5). Swal-QOL mean domain score in dysarthric patients was 82.9 ± 18.4 with six patients rated as dysphagic.

Twenty patients presenting significant dysphagia were identified using a cut-off of 70 in the Swal-QOL total domain score [16, 25]. For detailed patient characteristics, please refer to Table 1. Patients with dysphagia more often reported to regularly attend speech and occupational therapy than those without. Dysphagic patients were more fatigued and depressed than patients without dysphagia (*U* = 1656, *p* = 0.001 for FSS and *U* = 1784, *p* < 0.001 for BDI-II; compare Table 1).

**Fig. 2:**
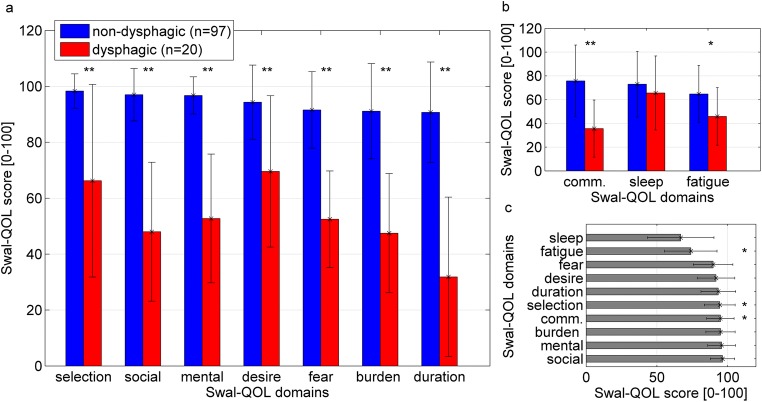
Swal-QOL domain scores for seven domains forming the sum score (**a**) and the other three (**b**). Similar sum score comparing non-dysphagic and control group (**c**; Rinkel et al. [26]; *p* = 0.06 (*t* test)), significant differences for domain scores are marked. Error bars = standard deviation; **p < 0.001; *p = 0.003

The Swal-QOL domain score differences between dysphagic and non-dyphagic patients are shown in Fig. 2. Scores were significantly different between groups for all domains except sleep (Mann-Whitney *U* test *p* < 0.003 for fatigue; *p* < 0.001 for the remaining domains). Duration of food intake and social function of eating were most compromised in the dysphagic group, whereas the desire to eat was less affected. Patients without dysphagia almost reached the same score as a healthy control group (Fig. 2c).

The dysphagia phenotype comprised all three phases of swallowing (pharyngeal, oral, and saliva-related) according to the 14 Swal-QOL items differentiating dysphagic disorders (data not shown). Coughing was the most commonly reported symptom in patients with dysphagia. No different phenotype was seen in dysphagic patients reporting speech disorders.

High scores on the Swal-QOL were associated with high mobility levels and high self-reported QOL while lower scores were associated with fatigue and depression. There was a positive correlation of Swal-QOL total domain score with mobility (*ρ* = 0.31, *p* < 0.001) and QOL (*ρ* = 0.39, *p* < 0.001) and a negative correlation with fatigue (*ρ* = − 0.39, *p* < 0.001) and depression (*ρ* = − 0.38, *p* < 0.001). There was no significant correlation of the Swal-QOL with etiology, age, gender, body weight, weight loss, disease duration, or nutritional habits.

### Body Weight and Nutrition

**Fig. 3:**
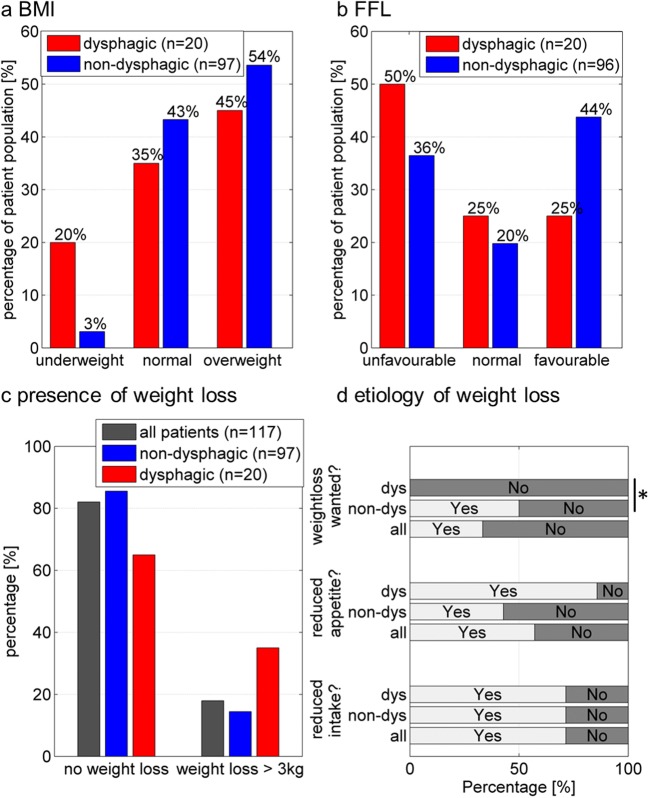
Weight (**a**) and nutrition (**b**) in patients. Dysphagic patients more often show underweight (**a** body mass index (BMI) < 18.5 kg/m) and classify weight loss (**c** > 3 kg in the last 12 months) as unwanted (**d**). dys = dyshagic; non-dys = non-dysphagic; *p = 0.02

Twenty-one patients (seven with dysphagia) reported weight loss of more than 3 kg in the past 12 months while 14 describing it as unintentional (Fig. 3c and d). All dysphagic patients in comparison to half of the non-dysphagic patients stated their weight loss to be unintentional (*χ*^2^ = 5.2, *p* = 0.02). There was no difference between groups regarding change in appetite or reduction of food intake (Fig. 3d).

Dysphagic patients more often reported a reduced food intake (45%) as compared to non-dysphagic patients (26%; *χ*^2^ = 4.2, *p* = 0.04) but body mass index (BMI) was similar in both groups. According to their BMI, seven patients (four of them with dysphagia) were classified as underweight, 49 (seven dysphagic) as normal weight, and 61 (nine dysphagic) as overweight (Fig. 3a).

Furthermore, no difference in nutritional habits in dysphagic as compared to non-dysphagic patients was verified among the 116 patients, for which FFL data was available (Fig. 3b). Scores for all patients ranged from 7 to 24 points with a mean of 14.5 ± 3.8. Forty-eight patients (41%) showed favorable, 25 (21%) normal, and 45 (38%) unfavorable nutritional habits. Dysphagic patients (*n* = 20) had a mean score of 13.1 ± 3.8; non-dysphagic patients (*n* = 96) had a mean score of 14.8 ± 3.7.

### Quality of Life

**Fig. 4:**
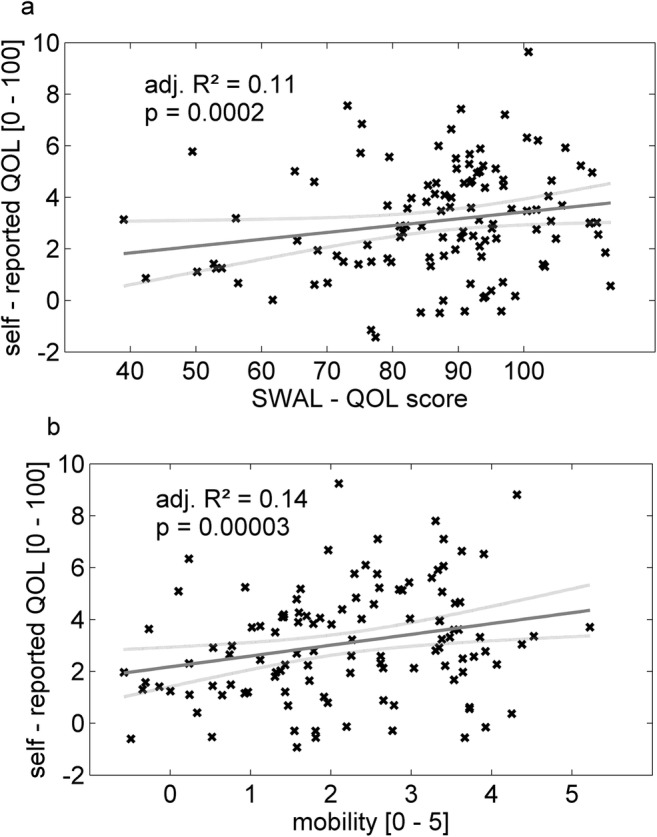
Multiple linear regression model for influence of dysphagia and mobility on self-reported quality of life (QOL) (0–10 numeric scale). Swal-QOL domain score (**a**) and mobility (**b**) explain 17% of variance in the subjective QOL. Adjusted *R*^2^ and *p* in the figure depict influence of the respective factor alone in the model

Dysphagic patients reported significantly lower quality of life scores (mean = 1.9 ± 2.2) than non-dysphagic patients (mean = 3.3 ± 2.4; *U* = 839.5, *p* = 0.01). Multiple linear regression analysis showed that greater mobility and higher Swal-QOL scores were associated with a higher quality of life (*p* = 0.00001, *F* = 13.16, adjusted *R*^2^ = 0.18, RMSE = 2.12; Fig. 4). No association for presence of fatigue or depression, disease duration, etiology, and nutritional habits (FFL) was seen in the regression analysis.

## Discussion and Conclusion

In our cohort, cerebellar ataxia patients hardly reported dysphagia as cardinal symptom in contrast to a considerable (17%) proportion identified as dysphagic by the Swal-QOL. Dysphagia was linked to unintentional weight loss, whereas nutritional habit changes did not become apparent. Apart from the well-established mobility decrease, we also found dysphagia to be associated with a reduced health-related quality of life (HRQOL) in patients with ataxia.

Among the 20 patients with dysphagia identified by the Swal-QOL, the only one deliberately reporting dysphagia as cardinal symptom was a patient with gastric tube and severe weight loss indicating a late stage of dysphagia. It would be useful to identify patients with dysphagia earlier, when unintentional weight loss is yet to be prevented. To this end, a standardized screening in the regular neurological care setting would be preferable before patients are referred to phoniatric consultation with videofluoroscopy. The widely varying prevalence of dysphagia reported in cerebellar ataxia reflects the underlying disease pathology and assessment methods. Using clinical case history, various dysphagia outcome scales, and/or videofluoroscopy, lower prevalence is being reported in pure cerebellar ataxia such as SCA 6 with 6–50% as opposed to complex cerebellar ataxia such as SCA 3 with 64–74% or Friedreich’s ataxia with up to 100% [1–3, 5, 27, 28]. Videofluoroscopy can detect even subclinical swallowing difficulties and thus leads to higher prevalence estimates [5]. Systematic evaluation of diagnostic procedures and treatment is lacking [29].

Additional to clinical examination in combination with clinical case history, a questionnaire such as the Swal-QOL may be useful as a screening tool in patients with cerebellar ataxia to identify dysphagia and its impact on HRQOL, direct patient care, and further examinations and prevent complications. A questionnaire in comparison to apparatus-assisted examination is less costly, independent of the clinical setting, better tolerated, and could guide further complex evaluation, e.g., videofluoroscopy to detect silent aspiration [28]. In a recent study, only 63% of dysphagic patients with Friedreich’s ataxia could be admitted to further evaluation by videofluorescopy, illustrating the difficulty of apparatus-assisted diagnostic procedure for dysphagia in ataxia patients even under study conditions [28]. In that study, dysphagia was evaluated in 60 patients with Friedreich’s ataxia using standardized oromotor assessment and videofluoroscopy, as compared to mainly late onset ataxia patients in our study. The authors focused on silent aspiration, which was found in 34% of patients undergoing videofluorescopy and was related to repeat-length and two subscores in oromotor assessment, but could not be predicted reliably [28].

Aspiration did not seem to affect swallowing-related quality of life, as no correlation with the Swal-QOL was reported and only 11 of 60 patients (18%) had a total domain score in the Swal-QOL below 70, which we used to identify dysphagic patients. Still, aspiration pneumonia represents one of the major factors for a reduced lifespan in hereditary cerebellar ataxia [13, 30]. The rate of patients experiencing recurrent bronchitis and pneumonia due to aspiration in a long-term study of 23 patients suffering from different forms of cerebellar ataxia was up to 50% and 13% of the patients died of aspiration pneumonia [31]. Thus, the correct diagnosis and recognition of dysphagia cannot be underestimated [29]. For detection of dypshagic symptoms, Swal-QOL and clinical case history should be considered and the Swal-QOL should be used as an additional tool to detect impact on HRQOL.

Dysphagia in neurodegenerative diseases such as cerebellar ataxia, Parkinson’s disease, and Huntington’s disease is known to cause malnutrition, weight loss, and reduced quality of life, but can also threaten a patient’s life [4, 8–11]. In Parkinson’s disease, an impact on quality of life was reported, and in Huntington’s disease, dysphagia correlated with disease severity and overall disability [32, 33]. In cerebellar ataxia, we could now show a correlation with HRQOL, fatigue, depression, and mobility. Disease severity, as rated by walking ability in our cohort, and Swal-QOL scores were shown to correlate with quality of life and with each other. Still, reduced mobility or gait instability were the most reported and most disabling symptoms in our cohort. Only mobility and dysphagia showed a clear association with HRQOL. We cannot exclude that a present impairment of HRQOL affects answers in the Swal-QOL and vice versa. A negative correlation of Swal-QOL score with fatigue and depression indices and a significant higher prevalence of pathologic fatigue and depression in dysphagic patients were seen. We thus controlled for concurrent presence of pathologic fatigue and depression in the regression analysis. In line with our results, reduced well-being linked to dysphagia in Friedreich’s ataxia has been reported [27]. Furthermore, Swal-QOL total domain score (including fatigue, sleep, and communication) has been shown to correlate with disease duration and severity in Friedreich’s ataxia [28]. We did not find a correlation with disease duration in our data and results remained unchanged when using the total Swal-QOL score including the above-mentioned domains. A possible explanation is the heterogeneity of our cohort with patients mainly suffering from SCA or sporadic ataxia and not a single patient suffering from Friedreich’s ataxia. A subgroup analysis for differences in dysphagia prevalence and correlations was not feasible due to small subgroup sizes.

In neurodegenerative disorders, lower body weight and weight loss have been shown to be correlated with rapid disease progression and worse functional outcome as well as a decrease in subjective quality of life [34–36]. Moreover, in children with ataxia telangiectasia, lower body weight was related to aspiration as assessed with videofluoroscopy [37]. Though weight loss is due to multiple factors, among them medication, motor impairment, intestinal factors, metabolic changes, and dysphagia, in our cohort, we could show unintentional weight loss to mainly occur in dysphagic ataxia patients. We did not find any differences in body mass index in dysphagic as compared to non-dysphagic patients, probably due to the subjective nature of data in a questionnaire. In line with our results, in a study with a long-term follow-up, nine of 19 patients with SCA 1 belonging to one pedigree reported weight loss [38]. Though in adult cerebellar ataxia, to our knowledge, no relation of weight loss and disease progression or outcome has been shown so far, unintentional weight loss and lower body weight in ataxia could be regarded as a potential risk factor for complications and should thus be prevented.

Furthermore, a tendency towards less favorable and more unfavorable nutritional behavior as defined by the German society for nutrition (DGN) in dysphagic patients was seen in our data. The relation of dysphagia and change of nutritional habits was statistically not significant. A larger study would be needed to confirm or reject this observation. The FFL has not been validated for patients with swallowing deficits but is well suited to assess nutritional habits and their deterioration in general. Still, additional questions concerning aspects of food texture and amount of food intake would be helpful for a patient population like ours.

In summary, we could show the Swal-QOL to be an additional useful and simple tool to identify patients with cerebellar ataxia suffering from dysphagia and rate the impact of dysphagia on subjective quality of life. Dysphagia may be a risk factor for unintentional weight loss and a tendency towards unfavorable nutritional behavior, probably leading to more severe complications such as malnutrition and recurrent infections.

Our work illustrates the necessity of further prospective studies to investigate the diagnostic work up and impact of correctly diagnosed dysphagia on disease progression, quality of life, and its association with disease severity and possible treatments.
